# “There’s nothing like a good crisis for innovation”: a qualitative study of family physicians’ experiences with virtual care during the COVID-19 pandemic

**DOI:** 10.1186/s12913-023-09256-3

**Published:** 2023-04-04

**Authors:** Lindsay Hedden, Sarah Spencer, Maria Mathews, Emily Gard Marshall, Julia Lukewich, Shabnam Asghari, Judith Belle Brown, Paul S. Gill, Thomas R. Freeman, Rita K. McCracken, Bridget L. Ryan, Crystal Vaughan, Eric Wong, Richard Buote, Leslie Meredith, Lauren Moritz, Dana Ryan, Madeleine McKay, Gordon Schacter

**Affiliations:** 1 Faculty of Health Sciences, Simon Fraser University, 8888 University Drive, Burnaby, British Columbia V5A 1S6 Canada; 2 Department of Family Medicine, Schulich School of Medicine and Dentistry, Western University, London, ON Canada; 3 Primary Care Research Unit, Department of Family Medicine, Dalhousie University, Halifax, NS Canada; 4 Faculty of Nursing, Memorial University, St. John’s, Newfoundland and Labrador, Canada; 5 Family Medicine, Faculty of Medicine, Memorial University, St John’s, Newfoundland and Labrador, Canada; 6 Department of Family & Community Medicine, Temerty Faculty of Medicine, University of Toronto, Toronto, ON Canada; 7 Department of Family Practice, Faculty of Medicine, University of British Columbia, Vancouver, British Columbia, Canada; 8 Department of Epidemiology and Biostatistics, Schulich School of Medicine and Dentistry, Western University, London, ON Canada; 9 Doctors Nova Scotia, Halifax, NS Canada

**Keywords:** Primary care, Family physicians, Virtual care, Telehealth, COVID-19, Canada

## Abstract

**Background:**

Prior to the pandemic, Canada lagged behind other Organisation for Economic Cooperation and Development countries in the uptake of virtual care. The onset of COVID-19, however, resulted in a near-universal shift to virtual primary care to minimise exposure risks. As jurisdictions enter a pandemic recovery phase, the balance between virtual and in-person visits is reverting, though it is unlikely to return to pre-pandemic levels. Our objective was to explore Canadian family physicians’ perspectives on the rapid move to virtual care during the COVID-19 pandemic, to inform both future pandemic planning for primary care and the optimal integration of virtual care into the broader primary care context beyond the pandemic.

**Methods:**

We conducted semi-structured interviews with 68 family physicians from four regions in Canada between October 2020 and June 2021. We used a purposeful, maximum variation sampling approach, continuing recruitment in each region until we reached saturation. Interviews with family physicians explored their roles and experiences during the pandemic, and the facilitators and barriers they encountered in continuing to support their patients through the pandemic. Interviews were audio-recorded, transcribed, and thematically analysed for recurrent themes.

**Results:**

We identified three prominent themes throughout participants’ reflections on implementing virtual care: implementation and evolution of virtual modalities during the pandemic; facilitators and barriers to implementing virtual care; and virtual care in the future. While some family physicians had prior experience conducting remote assessments, most had to implement and adapt to virtual care abruptly as provinces limited in-person visits to essential and urgent care. As the pandemic progressed, initial forays into video-based consultations were frequently replaced by phone-based visits, while physicians also rebalanced the ratio of virtual to in-person visits. Medical record systems with integrated capacity for virtual visits, billing codes, supportive clinic teams, and longitudinal relationships with patients were facilitators in this rapid transition for family physicians, while the absence of these factors often posed barriers.

**Conclusion:**

Despite varied experiences and preferences related to virtual primary care, physicians felt that virtual visits should continue to be available beyond the pandemic but require clearer regulation and guidelines for its appropriate future use.

**Supplementary Information:**

The online version contains supplementary material available at 10.1186/s12913-023-09256-3.

## Background

Prior to the COVID-19 pandemic, Canada lagged behind other Organisation for Economic Co-operation and Development countries in the uptake of virtual visits, particularly in primary care [1–3]. ‘Virtual visits’ or ‘virtual care’ in this manuscript refer to synchronous consultations with family physicians (FPs) via telephone or video conference. Pre-pandemic, virtual visits were provided by only 4% of FPs [3] and accounted for 0.15% of total billable services provided in Canada [4]. The drivers of this discrepancy in virtual visit uptake between Canada and other similar countries are complex and have not been well explored. Possible drivers include a lack of governance and compensation mechanisms for provincially-insured health services provided virtually; poor digital interoperability or connectivity across parts of the health care system; and licensure restrictions preventing physicians providing care to patients outside of their province of registration [1, 5].

The onset of the pandemic resulted in an unprecedented near-universal shift to virtual visits in primary care for everything except “essential urgent and emergency services” [6–9] to protect patients and clinicians from risks of COVID-19 infection. Provinces created (or modified) billing codes to allow clinicians to bill provincial health insurance plans for virtual visits (via telephone or videoconference) [10]. As the waves of the pandemic proceeded and health jurisdictions entered a pandemic recovery phase, the balance between virtual and in-person primary care visits has reverted somewhat [11]; however, it is unlikely to return to pre-pandemic levels due to ongoing demand from both clinicians and patients [12, 13].

### Clinician perspectives on virtual care

Case studies conducted before the pandemic in the United Kingdom and Norway found that introducing virtual care is a complex change that disrupts established clinical processes, practices, culture, and division of work [14–18]. In qualitative interviews, physicians raised concerns about privacy, safety, and litigation risk, as well as potential detriments to the quality of care provided to their patients [14, 17, 18]. In particular, many clinicians reported that technical challenges with video visits posed a barrier to their routine use [18, 19].

FPs also attributed challenges in implementing and sustaining virtual care during the pandemic to a lack of formal training and guidance for physicians providing virtual care [20, 21], inadequate reimbursement [22–25], and insufficient implementation supports [26–28]. Additionally, the absence of sufficient regulatory policies [29, 30] and emergence of virtual only walk-in clinics have been cited as concerns related to quality of care [22, 31].

Virtual modalities possess the potential to enhance quality of patient care and physician experiences, while minimising transmission risk of COVID-19 and other infectious diseases – particularly amongst those who are immunocompromised [32]. The shift to virtual care during COVID-19 expressly appreciated and sought to ameliorate the “Costs of Physical Contact” which, for patients, extends beyond the physical to include lost income from taking time off work, childcare, and transportation necessitated by in-person appointments [33]. This has been a particular concern for individuals living in rural, remote, and underserved communities with limited access to local health services for whom virtual modalities can improve access to primary care providers by removing inconvenient and sometimes arduous and costly travel requirements [5]. These varied potential benefits help to explain why, as we move beyond the COVID-19 pandemic, patients and FPs hope to have continued access to virtual modalities across Canadian health jurisdictions [12, 13].

Our objective was to explore Canadian FPs’ perspectives on the rapid move to virtual care during the COVID-19 pandemic to inform both future pandemic planning for primary care and the optimal integration of virtual care into the broader primary care context going forward.

## Methods

### Study design

This analysis was conducted as part of a larger study which sought to understand the formal and informal roles of FPs during the COVID-19 pandemic, and the barriers and facilitators FPs face in fulfilling those roles. The study consisted of multiple mixed-methods case studies that include a provincial policy scan, a chronology of FPs’ roles, and semi-structured interviews with FPs who provided primary care during the pandemic. A full study protocol has been published elsewhere [34]. Here, we report on an in-depth qualitative analysis of study data capturing the experiences of FPs with the transition to virtual care throughout the pandemic. All methods were conducted in accordance with and are reported following the Standards for Reporting Qualitative Research (SRQR) [35]. Additional analyses related to FPs’ impressions on the impact of virtualisation on their practice and their specific patient populations will be explored in a subsequent manuscript.

### Context

We conducted case studies in health regions in four Canadian provinces: Vancouver Coastal health region of British Columbia, Ontario Health West region, the province of Nova Scotia, and the Eastern Health region of Newfoundland and Labrador [36–39]. While these regions were pragmatically selected (i.e., the location of our research teams), they also exemplify the diversity of primary care structures and policy across Canada in their variation of primary care practice, remuneration models, and regional support structures, as detailed in our study protocol [34]. These regions also consist of both urban and rural communities and have experienced variations in the number of COVID-19 cases and the nature and duration of policy responses.

### Recruitment and data collection

We invited FPs to participate using physician hospital privileging lists, practice directories, word of mouth, social media, and snowball sampling. Interviews were conducted by members of the research team between October 2020 and June 2021. We used a purposeful, maximum variation sampling approach to achieve representation across characteristics of interest including career stage, model of practice and remuneration (core funding through fee-for-service or alternative payment model), gender, and community demographics [40]. We excluded FPs who did not hold an active practice licence or who worked solely in academic, research, or administrative roles. We also excluded students and post-graduate medical residents.

Prospective participants emailed study coordinators who in turn provided them with study information. Study coordinators (authors SS, RB, LMe, LMo, and MM) scheduled and conducted 45–60-minute interviews via Zoom or telephone, based on participant preference. To enhance rigour, we developed and pretested a semi-structured interview guide (Additional File 1) in consultation with our interdisciplinary study team of FPs, public health, and health systems experts. During interviews, we confirmed our understanding of participants’ responses to ensure accuracy and identify divergent experiences. Interviews were conducted in English, audio-recorded, and transcribed verbatim. We continued recruiting until no new themes emerged and there was sufficient data to support rigorous analysis (i.e., saturation).

### Analysis

We analysed transcripts using an inductive thematic approach [41]. Two members of the regional research teams reviewed each transcript and accompanying interviewer notes using a comparative analysis approach [41, 42] to identify initial themes and develop a preliminary coding framework for each region. Regional teams then met to compare their coding frameworks after coding the same transcript using their preliminary regional codes. In this cross-case meeting, we began developing a harmonised coding template by identifying overlapping regional codes, harmonising code names, descriptions, and the criteria for their application. This initial harmonised coding framework was then applied to a subset of transcripts from each region to refine and ensure coverage and applicability of the framework across a variety of FPs’ experiences. Throughout this process, codes evolved from broad and descriptive to more analytic through iterative content analysis [41]. Using the final harmonised coding framework, at least one researcher in each study region analysed the transcripts from their region using NVivo [43]. The focused analysis presented in this manuscript was led by a researcher with expertise in the expanding role of virtual modalities in the context of primary care in Canada.

### Ethics

We received ethics approval from the Behavioural Research Ethics Boards for Simon Fraser University and the University of British Columbia (through the harmonised Research Ethics British Columbia process), the Health Research Ethics Board of Newfoundland and Labrador, Nova Scotia Health Authority Research Ethics Board, and Western University Research Ethics Board. All participants provided written informed consent, recognising that their participation was voluntary. Responses were anonymised. We use participant codes throughout our presentation of findings, as well as an abbreviation of the participants’ region and core funding model – either fee-for-service (FFS) or alternative payment plan (APP, for all non-FFS models (e.g., salary, capitation)) – within which they work, to aid in contextualising their practice setting.

## Results

All 68 participants in the broader study discussed their experiences with virtual care during the pandemic and have been included in this focused analysis. A variety of different primary care practice and remuneration models were represented in the sample and are detailed alongside study participants’ characteristics in Table 1. Key themes arising from these interviews are summarised in Table 2.

**Table 1:** Participant characteristics [n (%)]

	British Columbia n = 15	Newfoundland & Labrador n = 12	Nova Scotia n = 21	Ontario n = 20	TOTAL n = 68
**Gender** ^a^					
Men Women	4 (36.4) 11 (63.6)	4 (33.3) 8 (66.7)	9 (42.9) 12 (57.1)	10 (50) 10 (50)	27 (39.7) 41 (60.3)
**Core Remuneration Type**					
Fee-for-Service Alternative Payment Plan^b^	6 (40) 9 (60)	5 (41.7) 7 (58.3)	7 (33.3) 14 (66.7)	4 (20) 16 (80)	22 (32.4) 46 (67.6)
**Hospital Privileges**					
No Yes	3 (20) 12 (80)	5 (41.7) 7 (58.3)	6 (28.6) 15 (71.4)	5 (25) 15 (75)	19 (27.9) 49 (72.1)
**Community Size** ^c^					
Rural Small Urban Urban Mix^d^	0 (0) 0 (0) 15 (100) 0 (0)	3 (25) 0 (0) 8 (66.7) 1 (8.3)	8 (38.1) 0 (0) 13 (61.9) 0 (0)	9 (45) 1 (5) 8 (40) 2 (10)	20 (29.4) 1 (1.5) 44 (64.7) 3 (4.4)
**Years in practice** (mean)	16.9	16.3	15.4	18.7	16.9

^a^ Gender was asked as an open-ended question

^b^ Alternate payment includes all non-fee for service or enhanced fee for service payment types as core remuneration

^c^ Rural ≤ 10,000 population, Small urban = 10,000–99,999 population, Urban ≥ 100,0000

^d^ Mix indicates FPs who practice in multiple communities of different size (rural, small urban, urban)

**Table 2:** Overview of key themes

**Theme 1 Implementation and evolution of virtual modalities during the pandemic** • Rapid transition to virtual care during initial stay-at-home closures • The evolving use of technologies (moving from video to telephone) • Striking the right balance between virtual and in-person care delivery
**Theme 2 Facilitators and barriers to implementing virtual care** • Prior experience with virtual modalities • Core remuneration model and billing structures • Technology and technical supports • Regulation and professional standards • Relationship-based care
**Theme 3 Virtual care in the future** • Expectations for virtual care beyond COVID-19 • Necessary changes to use, regulation • Physicians’ concerns

### Implementation and evolution of virtual modalities during the pandemic

Beginning in March 2020, jurisdictions across Canada declared public health emergencies and began imposing stay-at-home closures. Participants across jurisdictions echoed the need to minimise in-person care at this time, leading to the implementation of virtual modalities: “*… in that first time period, [from] like January to … early March is when I first remember noticing a shift on my schedule from in person to virtual”* [NS221 FFS]. Participants also noted how rapidly they were required to transform their work: “*We had to kind of pivot pretty quick to virtual care… as quickly as we could, right?”* [NL305 APP].

Some FPs explained how they were moving towards adopting virtual care before stay-at-home closures were introduced. These physicians promptly recognised the impact COVID-19 was likely to have and were proactive in minimising their clinic traffic and reducing transmission risks: *“we already started cutting down the personal visits in February [2020], unnecessary visits, and in March, we followed the guidelines, we tried to shift most of our patients to phone visits”* [ON118 APP].

As soon as governments enacted stay-at-home closures, provincial and regional medical health officers and physicians’ regulatory bodies encouraged FPs to provide all but essential care through virtual modalities, resulting in a drastic shift in how FPs provided care. In those early weeks and, in some cases, months of the pandemic, participants felt compelled to avoid in-person care. As one FP from Ontario noted, while “*80% of our work was done in-person*” pre-pandemic, the advent of COVID-19 saw their work patterns inverted: *“we switched to completely virtual and we’ve been totally virtual ever since”* [ON120 FFS].

After the initial response to the stay-at-home closures and the rapid conversion from in-person to virtual care, and as FPs gained experience and familiarity with virtual visits, the proportion of visits conducted virtually decreased. Participants noted that this re-balancing of virtual versus in-person visits was partly due to increased knowledge of COVID-19, increased access to personal protective equipment (PPE), changes to public health restrictions and FPs’ growing awareness of appropriate use of remote consultations (i.e., which patients and services required in-person visits):

*Over time, the balance of that has shifted to some degree, so depending on what’s going on in the community [in terms of COVID cases], we have sometimes moved to more in-person visits, depending on what the patient’s needs might be. And then just before Christmas [2020], kind of, heading back to more telephone visits, trying to delay any in-person visits that we possibly could. Now [January 2021], I would say that we’re about 50/50. I’d say my early part of January was more in-person, because we just could not delay putting off any more things. So, so probably a mix of both right now.* [NS208 APP]

As the pandemic progressed, participants gained more experience in triaging patients for in-person or virtual care, even in the absence of formal practice guidelines to inform their assessments:

*I think I ran four clinics that were completely virtual, after which it was like, ‘no, this doesn’t make sense, we need to start figuring out what’s appropriate for virtual, what’s appropriate for not virtual and how do we screen?’ And so, we started to build protocols for that.”* [ON114 APP].

Additionally, many participants shifted away from video-based care very early in the pandemic, moving to predominately phone-based care: *“I have not done a video visit since June [2020] and … I’ve done less than 10 video visits”* [NL306 FFS]. For many FPs, video interfaces proved challenging for them, their patients, or both – or simply provided no advantage over easier-to-navigate telephone visits:

*We dabbled in some video interviews, but those were harder because a lot of patients didn’t know how to do it; we didn’t know how to do it. So, we were excited about the video prospect initially, but I think we ended up doing a whole lot less video than we initially thought that we might.* [ON113 APP]

Through trial and error, physicians figured out how best to make virtual care work for them and their patients. One example that emerged repeatedly in the data was how physicians navigated assessing rashes/skin conditions. Several participants noted video calls were a challenging modality, largely due to the quality and movement of the image. For some FPs, a phone call combined with a still photograph was more appropriate:

*And then I found if it’s something I needed to look at, if it was like a rash that they wanted to show me, I found in fact, that if they took a photo and texted it to me, that actually worked better than video. So, a combination of photo, like texted photos and phone works the best, for me.* [BC408 FFS]

Others, however, felt that rashes (or anything else requiring a visual exam) required an in-person assessment: “*I can’t look at your rash over the phone or your computer webcam that I can’t see anything out of. So you would have them come in [for an in-person visit]”* [NS221 FFS].

### Facilitators and challenges to implementing virtual care

#### Prior experience with virtual modalities

Participants experienced the implementation of virtual care differently depending on their prior use of virtual modalities and whether their clinics had the necessary infrastructure already in place. For FPs with no prior experience conducting remote assessments, the transition to virtual care introduced several challenges and required a restructuring of their practice and workflows:

*So, my secretary wasn’t delivering me the charts of patients that I’m going to talk next day. So, our office was very old-school office, we didn’t have [electronic medical records (EMR)]; I never had training for EMR. So, we all had pen and paper and charts and patients- that’s it. So, I need to learn how to go and check the medication, how to go and find the patient’s prescription because I want to order prescriptions and I didn’t have the actual chart. I had to learn how to go and find the consult or do this or do that. And, then later, when you want to do the virtual visits in the room, like how do I book my patients? How do I set up my devices to them? And it was learning how to basically call the patients, not through the [video-telephone platform] or the texting, but through the hospital setting that they give you all the confidentiality that is needed for the appointment. So, all of that need to be learned along with the fact that I had to fax the stuff and I had to deal with all of my personal life and everything from March 18th- that was like the biggest shock. Because, as I said, all I used to do was writing, writing, writing, but now everything was virtual for me; everything was over the computer.* [NL312 FFS]

Conversely, practices with virtual care experience and existing infrastructure required less drastic adaptation:

*… we had a flow already developed for how to do virtual care because we’ve been doing it. So, it was a matter of just kind of… flipping our services so that it was more virtual than in-person … the proportion flipped. But in terms of our comfort with using the technology, for example, that was already there.* [BC415 FFS]

#### Core remuneration model and billing structures

Clinicians remunerated in primarily APP models were able to more easily transition to virtual care than FFS clinicians who, in the majority of provinces, were unable to bill provincial health insurance for virtual consultations prior to the pandemic:

*… – because of our population-based funding model – you know, an encounter is an encounter, it doesn’t matter if it’s… by phone or a refill straight to the pharmacy or a discussion with a specialist colleague. And we had been doing that for the last 20 years.* [BC407 APP]

Conversely, in Ontario, FFS physicians were initially required to use the Ontario Telemedicine Network (OTN) for virtual visits [44, 45], and some respondents found that system was “*always kind of glitchy”* [ON 120 FFS], “*confusing and…[not] very user friendly”* [ON118 APP]. One participant noted that their “*95-year-old [patients] couldn’t figure out how to do OTN*”, and that:

*…it doesn’t work very well. Sometimes it works great, but what OTN does right now is … often you’re speaking and your video are out of sync. And so, it makes it very difficult to really have a conversation.* [ON106 FFS]

The rapid introduction of fee codes to remunerate FFS physicians for virtual care provision was a prominent facilitator discussed across several regions:

*One of the things I think was a success was how nimble [the Department of Health and Wellness] and [Nova Scotia Health] were in getting our virtual care billing set up. I think that was within days, if I remember correctly.* [NS213 FFS]

However, this experience was not universal, and physicians in some provinces reported lengthy delays in receiving payment under these new codes [46]: “*[it took] 3 or 4 months to get paid…. for [physicians on FFS] versus in family health team or roster-type situations [i.e., capitation-based funding]”* [ON115 APP].

#### Technology and technical supports

An FP in Newfoundland and Labrador noted the utility of their EMR systems that pre-dated COVID-19 for facilitating the shift to virtual care by allowing access to patient information remotely, or incorporating virtual visits seamlessly into exiting EMR systems: “*I really don’t know how we would have managed to do virtual care if we didn’t have the EMR”* [NL307 FFS]. However, adapting EMRs to incorporate video did come at additional cost: “*The core functionally of the EMR is a shared pay agreement, partly paid by the provincial government, partly paid by the docs. […] But that additional video-conferencing abilities, 100% paid by the docs.”* [NL308 APP].

The clinics that did not have the technology in place had to seek out the requisite programs to support video consultations as a supplement to telephone visits on top of their regular work:

*We had to do it as an add-on to all of what we were already doing, which is to research all the options, select one, purchase one, implement one, test one … and that’s a lot of extra work that we had to do just to be able to add on… the video virtual care.* [BC401 APP]

Other participants described the lack of support they felt and the barriers they encountered when they were instructed to convert to a predominately virtual practice at the outset of the pandemic. Some of these barriers were in relation to the costs – both time and financial – associated with researching, selecting, and implementing virtual modalities:

*We also were getting our head around, from a clinic point of view, how we would virtually connect with our patients. We use [video-conferencing platform], and so we, we did a couple of things, we looked at how we would do it from an audio point of view and also how we would do it from an audio-visual point of view. So, we did connect with [telecommunication company] and got some software that would allow for the audio-visual connection, because that had a degree of privacy that [other video-conferencing platforms] didn’t offer. And then we had to come up with […] a triage mechanism for which we would deal with our patients. So, audio-wise, audio-visual-wise, in-person.* [NL 301 APP]

Some participants noted the technical support they received from their health organisations and/or provincial government: “*[the regional organisation] rolled out a virtual help desk type thing. They have an IT guy specific for trying to get a [video-conferencing platform], up and running and downloaded and getting us accounts and thankfully”* [BC404 APP].

#### Regulation and Professional Standards

For some FPs, assurances from regional professional organisations helped to abate their concerns about privacy and confidentiality of providing care virtually:

*We were allowed to do medicine over [video-conferencing platform] and [video-telephone platform] basically, where [the medical regulator] said that that’s okay in terms of privacy. So, I didn’t have to do my own privacy assessments of things and turn them into my privacy policy, so that was very helpful.* [BC402 FFS]

Other participants, however, still had concerns about ensuring that patients understood potential risks. Referring to privacy concerns around patients sending pictures or health information via email or text message to facilitate better phone consultations, participants noted the disclaimers they provided to patients about the insecurity of sending medical information by email, leaving the choice up to the patient based upon their comfort.

The lack of training in virtual care appeared also to be exacerbated by the lack of guidance from physicians’ regulatory colleges, with participants noting, *“the challenge of learning how to do telephone medicine*”, in particular that *“there are medical legal issues of what you have to document”* [NS205 APP]. This aspect of implementing virtual care left many FPs uncertain of how to uphold their professional obligations and standards while providing a type of care with which they had limited or no familiarity: *“So, we never had any guidance as to what to do at the clinics, who to bring in, what our obligations were, when we would get PPE, would we get paid even…”* [NL311 FFS].

#### Relationship-based care

Participants also highlighted the importance of their pre-existing relationships with patients. Given the scarce use of virtual modalities prior to COVID-19, transitioning to virtual care was made easier by long-term relationships because their familiarity with their patients allowed FPs to transition to virtual care more smoothly and assess more easily when an in-person or virtual visit was more appropriate:

*I’m grateful that I’ve had long-standing relationships with those patients, some of them I’ve known for 15 years and I think that makes [delivering care virtually] easier. I would really struggle to deliver the same kind of connection … if I had to see somebody for the first time over the phone or over [the video-conferencing platform].* [BC404 APP]

### Virtual care in the future

Regardless of their personal use and perspectives on virtual care, nearly all participants believed that virtual models of care will be a permanent part of primary care delivery after the pandemic:

*I suspect that [virtual care is] going to be a permanent fixture now, because it worked so well. …We each now, in our clinic, have a half-day virtual clinic. We’re pretty well back to normal in the clinic seeing patients, but each of us has taken at least a half-day to do telephone calls.* [NL305 APP]

In addition, participants noted the need to maintain or enhance virtual care billing codes that were introduced during the pandemic: *“Well, we just need to keep a billing structure for phone and video visits with people; we should not let that go”* [NS201 FFS]. While most participants expressed confidence that virtual care would remain beyond the pandemic, they noted that the current virtual care billing codes were intended to be temporary pandemic measures.

FPs were supportive of virtual care – telephone visits in particular – remaining part of regular practice beyond the pandemic; however, many physicians commented on the need for “*clear guidelines and restrictions”* [NS221 FFS], as well as improved supports to ensure good quality care and an appropriate balance between virtual and in-person visits. Virtual care *“needs to be implemented very thoughtfully and carefully, and probably adjusted as things go along”* [NS204 FFS]. Many physicians pointed to the potential for misuse, should such guidelines and regulations not be established. One participant noted their concerns about the potential for excess reliance or overuse of virtual care, and specifically the trade-off between accessibility and continuity of care related to stand-alone virtual ‘walk-in’ practices: “*And specifically, though, for continuity of care situations, because the virtual care blowing up too – I always call it McDonald’s medicine – is not always the best medicine. So, continuity of care being important with that virtual medicine accessibility”* [ON111 APP].

## Discussion

This paper has highlighted FPs’ perspectives on and experiences with the implementation and use of virtual care during COVID-19, as well as on the ongoing integration of virtual care within regular primary care practice. FPs, most of whom had no or very limited experience delivering care virtually, were required to adopt virtual visits rapidly to support patients and limit the spread of infection in the changing pandemic environment. This transition represented a significant change to workflows and clinical practice [14–18]. It also required a substantial investment of time and money as physicians had to learn about different technologies, regulatory and privacy requirements, vendors, as well as how to adapt their practices to those modalities. FFS physicians in Ontario in particular reported frustrations with using the required OTN service, and delays in receiving payment for care they provided virtually [44–46].

The extent to which FPs had prior experience conducting virtual visits, necessary infrastructure already in place, and technological troubleshooting supports (offered through regional structures such as Health Authorities) strongly influenced the ease with which they transitioned to virtual care during the pandemic. FPs with less support appeared to experience more challenges. FPs noted that the absence of regulatory or clinical guidance on the use of virtual visits was also a challenge. These findings are consistent with existing literature, which has identified that a lack of infrastructure [20] and absence of regulatory policies [29, 30] are ongoing challenges to effective virtual care implementation, reinforcing the need for structured policy development in this area.

The shifts in the relative balance between virtual and in-person visits during different time periods in the pandemic (from a preponderance of virtual visits during closure periods to a return to in-person visits between COVID-19 waves) reflects complementary analyses of changes in physician billing patterns over the course of the pandemic [11, 47]. Additionally, interview data with physicians highlight the shift from video to telephone visits soon after their initial forays with the former, as clinicians experienced more technological challenges and limited additional benefit with video relative to telephone calls, consistent with existing research [48]. Additional research is needed into when and how video interfaces can result in improved quality of care, efficacy, and patient satisfaction to support the development of evidence-informed regulatory and remuneration guidelines for telephone versus video virtual care interfaces.

Echoing national survey data [13], the physicians we interviewed had generally positive opinions about the continued inclusion of virtual models of primary care beyond the pandemic but identified the pressing need for regulatory policies, technical supports, permanent billing codes [22–25], clinical best practice guidelines and decision supports [29, 30], and education and training to promote equitable, accessible, and appropriate virtual care. Physician associations and provincial policymakers should collaborate on and prioritise the development of such policies and practice and implementation guidelines. Additionally, FPs highlighted the importance of longitudinal patient-FP relationships and continuity of care in the delivery of care through virtual modalities. These unique qualities of family medicine have been previously identified as important supports in mitigating the negative impacts of rapid shifts in the availability and delivery of care during the pandemic [49–51]. Notably, recent updates in Ontario to the 2021–2024 Physician Services Agreement have introduced permanent virtual care billing codes to the Ontario Health Insurance Plan, replacing the temporary codes introduced during the pandemic. These codes distinguish between care provided in the context of an existing longitudinal patient/provider relationship (i.e., Comprehensive Virtual Care Services) and that which can be reimbursed outside of such care contexts (i.e., Limited Virtual Care Services) [52, 53]. Additional research is needed to evaluate the impact of such policies on patient access to care and physician work experiences.

The provision of virtual care became an expected role for FPs during the COVID-19 pandemic [54] and is part of a multi-pronged strategy to minimise exposure risks for patients, FPs, and clinic staff [55] while mitigating PPE needs associated with in-person consultations [56] during a pandemic. Future research should evaluate the effectiveness of increasing the use of virtual modalities in primary care during flu seasons and for clinically vulnerable patients to reduce infection risks.

### Limitations

This study focused on FPs roles during the pandemic within health regions in four Canadian provinces. While our sample of FPs was intentionally heterogenous, reflecting a variety of practice and remuneration models, practice locations, and COVID-19-related roles and experiences, we did not explicitly compare across particular practice or demographic groups. Our sample had a large number of physicians on alternative payment models relative to the general population of FPs. While virtual care was not the primary focus of our study, discussions of virtual care emerged organically during interviews in the context of pandemic roles and responsibilities. Further research is needed to understand the use of virtual care and its facilitators and barriers outside the context of the pandemic [51]. Interviews were conducted between October 2020 and June 2021 and may not reflect experiences related to the use of virtual care later in the pandemic. Finally, our interview data may be subject to biases such as social desirability [57] and response bias [58].

## Conclusion

Adopting virtual models of care during the COVID-19 pandemic was a source of a considerable increase in workload for FPs, particularly for those who had limited prior experience delivering care virtually. While FPs were positive about the integration of virtual care as a permanent fixture in primary care delivery, varied experiences during the pandemic underscore the need for training, guidance, and regulations to ensure equitable access and quality of patient care. Existing longitudinal relationships between physicians and patients, which is a hallmark of family medicine, facilitated successful transitions to virtual care.

## Electronic supplementary material

Below is the link to the electronic supplementary material.

Supplementary Material 1

## Data Availability

The datasets generated and analysed during the current study are not openly available to maintain the anonymity and confidentiality of participants but may be available from the corresponding author on reasonable request.

## Abbreviations

APP: Alternative payment plan
EMR: Electronic medical record
FFS: Fee-for-service
FP: Family physician
PPE: Personal protective equipment
OTN: Ontario Telemedicine Network
